# Role of serum complement C3 and C4 on kidney outcomes in IgA nephropathy

**DOI:** 10.1038/s41598-024-65857-w

**Published:** 2024-07-13

**Authors:** Edoardo Tringali, Daniele Vetrano, Francesco Tondolo, Federica Maritati, Benedetta Fabbrizio, Gianandrea Pasquinelli, Michele Provenzano, Gaetano La Manna, Olga Baraldi

**Affiliations:** 1 Department of Medical and Surgical Sciences (DIMEC), Alma Mater Studiorum University of Bologna, Bologna, Italy; 2 Nephrology, Dialysis and Kidney Transplant Unit, IRCCS Azienda Ospedaliero-Universitaria di Bologna, Bologna, Italy; 3 Pathology Unit, IRCCS Azienda Ospedaliero-Universitaria di Bologna, Bologna, Italy; 4 Department of Pharmacy, Health and Nutritional Sciences, University of Calabria, Rende, CS Italy; 5 Nephrology and Dialysis Unit, Santa Maria delle Croci Hospital-Ravenna, AUSL Della Romagna, Ravenna, Italy

**Keywords:** Complement, C3, C4, IgA nephropathy, MEST, Glomerulonephritis, Nephrology, IgA nephropathy

## Abstract

IgA Nephropathy (IgAN) is the most prevalent glomerular disease worldwide. Complement system activation is crucial in its pathogenesis. Few studies correlated serum C3 and C4 with disease activity and prognosis. This retrospective study investigated the prognostic value of serum complement at the time of diagnosis in patients with IgAN. Specifically we evaluated whether adding serum C3 and C4 levels to established predictive models-one based on variables related to chronic kidney disease (CKD) progression and another incorporating variables from the International IgA Prediction Tool (IntIgAPT)-enhances the accuracy of outcome prediction. A composite renal outcome was defined as 50% decline in eGFR or onset of kidney failure. 101 patients were stratified according to baseline C3 levels in three groups (Low, Medium and High). During a median follow-up of 54 months, the Low group exhibited higher incidence of primary outcome (16.3 events vs 2.9 and 1.7 events × 100 pts/year, p = 0.0026). Model-1 (M1), consisting of CKD progression variables, and Model-3 (M3), comprising IntIgANPT variables, were implemented with baseline C3 and C4 to create Model-2 (M2) and Model-4 (M4), respectively. M2 demonstrated better predictive performance over M1, showing higher discrimination (lower AIC and BIC, higher C-index and NR2). Similarly, M4 outperformed M3, showing enhanced outcome prediction when C3 and C4 levels were added. Implementation of serum C3 and C4 can enhance prediction accuracy of already-validated prognostic models in IgAN. Lower C3 and higher C4 levels were associated with poorer prognosis, highlighting a more 'Complement-Pathic' subset of patients.

## Introduction

IgA Nephropathy (IgAN) is the most common glomerular disease worldwide, representing approximately 22% of diagnoses in Europe and 11% in North America^1^.

Clinical manifestations, disease course and response to immunosuppressive treatment are extremely heterogeneous, with a 10-year risk of kidney failure (KF) ranging from 5 and 60%^2,3^.

According to the current guidelines^4^, following a biopsy-proven diagnosis of IgAN, disease prognosis should be assessed using the MEST-C system. This histologic score evaluates lesion such as mesangial (M) and endocapillary hypercellularity (E), segmental sclerosis (S), interstitial fibrosis-tubular atrophy (T) and crescents (C)^5^. Such histological findings have been recently incorporated in a prognostic tool (International IgAN Prediction Tool [IntIgAPT]) together with clinical and biochemical variables collected at the time of biopsy. This system, validated in more than 4000 patients, may predict the risk of disease progression at 5 years (50% of estimated Glomerular Filtration Rate [eGFR] decline or KF), helping subsequent treatment strategy^6–8^. However, neither this calculator nor the MEST-C score alone can be used to determine the likely impact of any treatment regime on disease course. Thus, treatment strategy is based on the severity of proteinuria and estimated eGFR.

Enhancing risk stratification is an urgent need in nephrology research. This effort can be accomplished by the development of reliable prognostic biomarkers^9^.

Complement system (CS) activation is crucial in IgAN pathogenesis. Both the alternative (AP) and lectin pathways (LP) can be activated, resulting in the production of anaphylatoxins, as well as the formation of the cytolytic membrane attack complex. This leads to the stimulation of mesangial cells to produce inflammatory mediators and matrix proteins^10,11^. The CS can be activated both locally, as demonstrated by the presence of C3 deposits alongside IgA in over 90% of patients, and systemically, with elevated levels of subproducts of C3 found in affected patients^12^. Until now, the role of CS has primarily been explored through histological, genetic and biochemical investigations, focusing on the byproducts of cascade activation and detectable metabolites in tissues, blood, and urine. To date, only few studies have correlated serum C3 and C4 fractions with disease activity and prognosis^13,14^.

Given the pivotal role of CS in disease initiation and progression, we share findings from a longitudinal IgAN cohort study, examining the potential advantages of incorporating serum C3 and C4 levels at the time of diagnosis into established prognostic models for risk stratification.

## Materials and methods

### Patients and treatments

We retrospectively reviewed clinical records of patients with a histology-proven diagnosis of IgAN referred at our unit (Sant’Orsola University Hospital, Bologna) from Jan-2009 to Dec-2022.

Inclusion criteria were: (I) renal biopsy scored according to Oxford MEST-C scoring system; (II) availability of medical history, blood pressure (BP) measures, data related to renal function (serum creatinine and eGFR), urinary exams (urine biochemistry, 24-h proteinuria [uProt]), serum protein profile (serum albumin and total protein), serum complement levels (C3 and C4), and immunoglobulin (Ig) levels of IgA, IgG, and IgM at the time of renal biopsy; (III) follow up duration of at least 12 months; (IV) at least 18 years at diagnosis. Exclusion criteria were: (I) KF at diagnosis, (II) over-imposed nephropathy.

Treatment was administrated according to current clinical practice and guidelines recommendations. Optimized first-line included management of BP and other cardiovascular risk factors, lifestyle modification and maximally tolerated dose of Renin–Angiotensin–Aldosterone-System inhibitors (RAASi) and Sodium/Glucose Cotransporter-2 inhibitors (SGLT-2i). High-risk patients (i.e. severe proteinuria and/or impaired eGFR) were offered a personalized course of immunosuppression therapy, for example steroids, both systemic (Manno or Pozzi regimens) and local (Budesonide).

All patients received regular follow-up care at our outpatient nephrology clinic, which included blood and urine tests, as well as comprehensive clinical evaluation.

The study was performed in accordance with the declaration of Helsinki and the protocol was approved by the ethical committee of the Sant’Orsola University Hospital of Bologna (Protocol number 420/2018/Oss/AOUBo). Patients provided informed consent for study participation.

### Data collection

At the time of admission for kidney biopsy, a thorough medical and pharmacological history of the patients was conducted. Additionally, BP measurements were taken using a standardized office method. Furthermore, patients underwent both blood and urine laboratory tests. All data were collected in electronic spreadsheet. Diagnosis-subsequent pharmacological treatment regimen, with particular focus on supportive—(i.e., RAASi) and immunosuppressive treatment (e.g., steroids)—were also obtained.

### Kidney biopsies

Kidney biopsies were performed in the suspicion of kidney disease (e.g., urinary abnormalities and/or functional impairment) after written consent of the patient and in accordance with current clinical practice (percutaneous ultrasound-guided approach). Histological samples were processed and evaluated by local nephropathologist under light microscopy, immunofluorescence, and electron microscopy.

### Study aim

The main aim of this retrospective observational single-center study was to investigate the prognostic value of serum C3 and C4, when added to the already known prognostic IgAN models, including IgANPt. The primary outcome used for model computation was a kidney composite outcome, defined as a 50% decline in eGFR and/or the onset of KF.

Secondly, we investigated the interaction of serum complement with other baseline clinical features on the primary endpoint.

### Statistical analysis

Descriptive statistics were reported as means ± standard deviations (SD) or median and interquartile range (IQR) for continuous variables, according to distribution. Categorical variables were reported as percentages (%).Patients were stratified according to their baseline serum C3 levels into three categories: Low [< 90 mg/dl], Medium [90–140 mg/dl], and High [> 140 mg/dl], determined by tertiles of the variable distribution in our sample. These cut-offs matched the laboratory levels of serum C3 abnormalities in our Centre. Comparison between groups were tested by means of ANOVA or Kruskal–Wallis test for continuous variables according to distribution. Categorical variables were compared using the Chi-squared test. Linear associations between C3, C4 and other continuous baseline variables involved in IgAN pathophysiology were plotted graphically and tested with Pearson coefficients. Multivariate linear regressions were used to assess correlation between C3 and C4 with eGFR. The slope of the regression line (beta coefficient, [β]) and its corresponding p-value were used to assess the strength and significance of the linear association. Median follow-up was computed by means of inverse Kaplan–Meier approach. Patients were followed until the onset of the primary outcome or until the last follow-up visit in nephrology clinic. Patients lost to follow up were right censored at the time of the last outpatient visit. For the survival analysis we observed sufficient events to compute the incidence rate of outcome. To assess the additional prognostic value of serum complement in comparison with the ‘already used models’ we adopted a logistic regression approach.

As ‘already used models’, we selected: a first one used in clinical practice based on classical variables of kidney disease progression namely age, BP, eGFR, proteinuria^15^; a second one including the variables of the IntIgAPT. For each comparison, we first built a model without C3 and C4 and then added these two variables to build a new model. We computed the measures of goodness of fit: Akaike Information Criterion (AIC) and Bayesian Information Criterion (BIC), Nagelkerke R^2^ test (NR^2^) that depicts the % explained variation of the outcome based on the set of variables included, and Likelihood Ratio Test (LRT); discrimination with the c-index.

A p-value of < 0.05 was considered statistically significant.

Statistical analysis was performed using R version 4.0.3 (R Core Team, 2020).

## Results

### Patients characteristics stratified by C3 groups

101 patients were included in the cohort. 90% were Caucasian and 65.3% were male with a mean age of 42.6 years. As reported in Table 1, mean eGFR and median proteinuria levels at baseline were 70.4 ml/min/1.73 m^2^ and 1.04 g/24 h respectively. Mean serum C3 was 114.9 mg/dL.

**Table 1:** Basal characteristics stratified by serum C3.

	High ≥ 140 N = 14	Medium 90 -140 N = 72	Low ≤ 90 N = 15	Overall N = 101	p-value*
Male gender, n (%)	12 (85.7%)	46 (63.9%)	8 (53.3%)	66 (65.3%)	0.185
Age at biopsy years (± SD)	46.29 (9.45)	41.15 (14.44)	46.13 (13.01)	42.60 (13.73)	0.249
Hypertension, n (%)	12 (85.7%)	37 (51.4%)	11 (73.3%)	60 (59.4%)	**0.028**
SBP, mmHg (± SD)	133.57 (14.99)	122.83 (16.15)	129.00 (14.04)	125.26 (16.06)	**0.044**
DBP, mmHg (± SD)	80.71 (9.17)	76.79 (11.08)	79.33 (8.84)	77.72 (10.54)	0.365
Diabetes, n (%)	3 (21.4%)	3 (4.2%)	1 (6.7%)	7 (6.9%)	**0.04**
CVD, n (%)	3 (21.4%)	7 (9.7%)	3 (20.0%)	13 (12.9%)	0.324
Smoke, n (%)					
Ex	2 (14.3%)	3 (4.2%)	1 (6.7%)	6 (5.9%)	0.396
Current	2 (14.3%)	7 (9.7%)	2 (13.3%)	11 (10.9%)	
eGFR, mL/min/1.73 m^2^ (± SD)	68.64 (23.96)	75.67 (36.17)	47.00 (31.81)	70.44 (35.33)	**0.015**
uProt, g/24 h [IQR]	1.81 [1.15, 4.10]	0.75 [0.30, 1.60]	1.46 [0.88, 3.18]	1.04 [0.38, 1.90]	**0.004**
Tot. protein, g/L (± SD)	6.91 (1.23)	6.72 (0.68)	6.41 (0.91)	6.70 (0.78)	0.437
Albumin, g/L (± SD)	3.98 (0.84)	3.95 (0.70)	3.59 (0.72)	3.91 (0.72)	0.410
C4, mg/dL (± SD)	40.71 (8.65)	33.56 (9.48)	27.40 (10.64)	33.63 (10.11)	**0.001**
IgG, mg/dL (± SD)	1086.36 (325.43)	985.40 (267.29)	956.20 (363.41)	995.45 (291.51)	0.429
IgA, mg/dL [IQR]	263.50 [220.50, 460.25]	302.00 [236.00, 371.00]	273.00 [217.50, 411.00]	294.00 [229.75, 377.75]	0.975
IgM, mg/dL (± SD)	86.43 (38.67)	100.49 (43.30)	119.33 (65.64)	101.39 (47.19)	0.166
CD4/CD8 ratio (± SD)	1.74 (0.64)	1.72 (0.60)	1.78 (0.52)	1.73 (0.59)	0.907
Immunosuppression, n (%)	12 (85.7%)	49 (68.1%)	11 (73.3%)	72 (71.3%)	0.479
Composite renal outcome, n (%)	1 (7.1%)	6 (9.2%)	5 (33.3%)	12 (12.8%)	**0.015**
Time to outcome months (± SD)	54.28 (13.08)	45.57 (34.04.)	27.53 (19.34)	43.86 (30.75)	**0.040**

*SD* standard deviation, *IQR* interquartile range, *SBP* systolic blood pressure, *DBP* diastolic blood pressure, *CVD* cardiovascular disease, *eGFR* estimated glomerular filtration rate, *uProt* 24 h urinary protein.

*p-value refers to comparisons of variables between C3 risk categories (high, medium, low).

Significant values are given in bold.

Stratifying by serum C3 levels (Table 1), we didn’t find significant differences in gender or age. High and Low groups had a significantly higher prevalence of hypertension (High 85.7%, Medium 51.4%, Low 73.3%, p = 0.028) with a significant higher systolic blood pressure (SBP) (mean High 133.6, Medium 122.9, Low 129.0, p = 0.044). There were no differences in the rates of CVD or smoking habit. The High group had a higher percentage of diabetes than Medium and Low (High 21.4%,Medium 4.2%, Low 6.7%, p = 0.04). The Low group had lower values of eGFR at baseline (High 68.6, Medium 75.7, Low 47.0 ml/min/1.73 m^2^, p = 0.015), while the Medium group had lower values of uProt (High 1.81, Medium 0.75, Low 1.46 g/24 h, p = 0.004). There were no differences in serum total protein, albumin, IgG, IgA, IgM, or CD4/CD8 lymphocytes ratio. There was a significant difference in serum C4 values, which seemed to decrease, switching from High to Medium to Low (High 40.7, Medium 33.6, Low 27.4 mg/dL, p = 0.001).

### Associations between serum C3 and C4 and baseline variables

**Figure 1:**
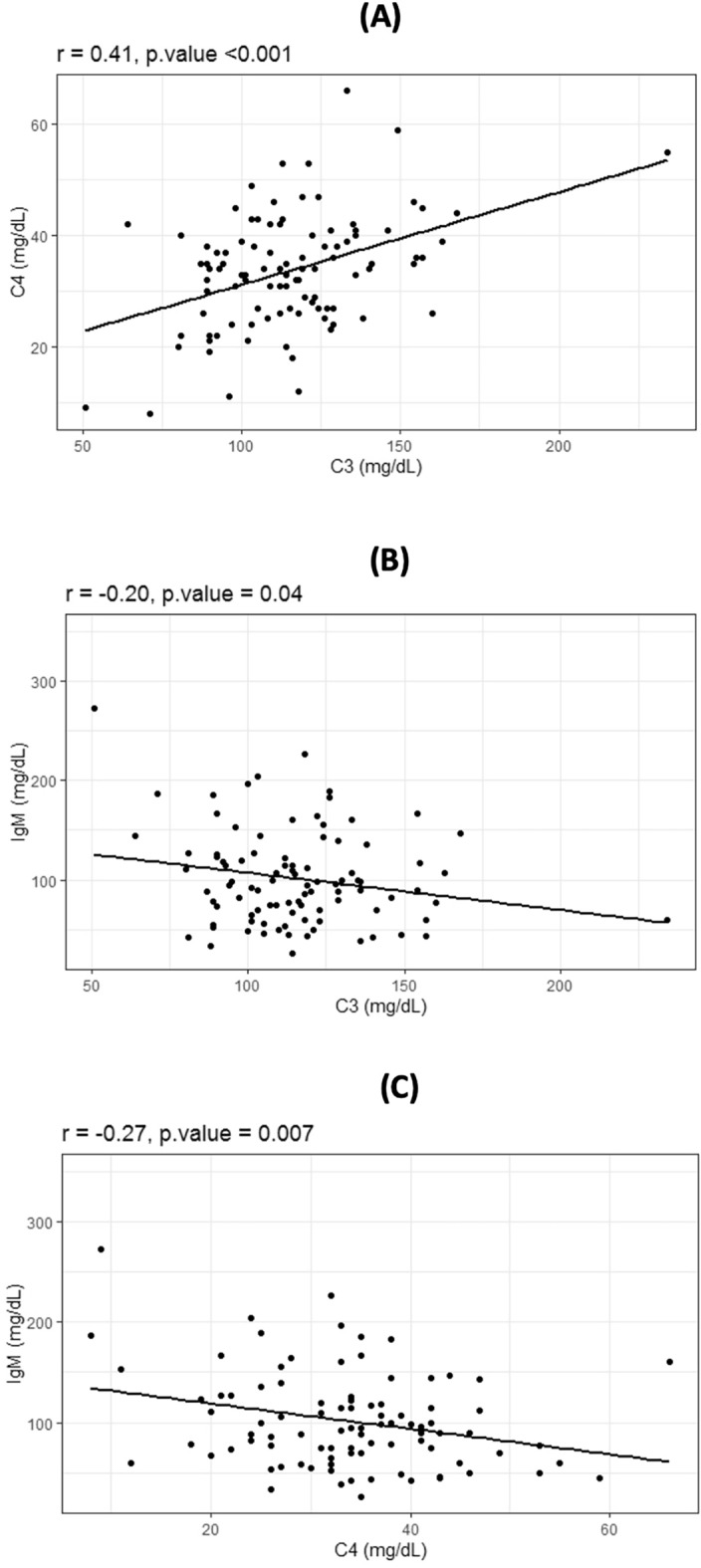
Results from the correlation analysis showing the relationship between baseline variables. In particular, (**A**) shows the positive relation between C3 a C4. (**B**) The negative relation between C3 and IgM and panel C shows the negative relation between C4 and IgM.

Baseline serum C3 and C4 levels were positively correlated (r = 0.41, p < 0.001) (Fig. 1). There were no associations between C3 and levels of IgA, IgG (Fig. S1—Supplementary). Instead, C3 exhibited negative correlation with IgM (r = − 0.20, p = 0.047). C4 levels were not associated with IgA, IgG, but were negatively correlated with IgM (r = − 0.27, p = 0.007). In multivariate linear regression model, both C3 (β = 0.30, p = 0.05) and C4 (β = − 0.76, p = 0.045) were associated with baseline eGFR (Table S1—Supplementary).

### Follow-up and prognosis according to baseline complement values.

**Figure 2:**
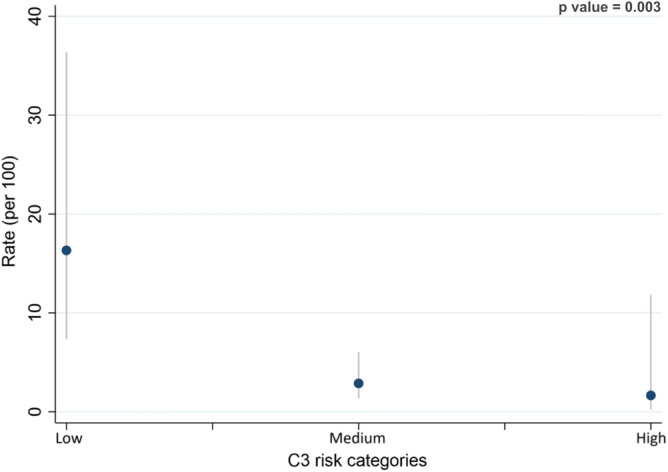
Incidence of outcome expressed as incidence rate ratio (100 patients[pts]/year) stratified by baseline C3. Low group had a higher incidence, 16.3 events (95% CI 7.3–36.3) compared with Medium (2.9 events 95% CI 1.4–6.1) and High group (1.7 events 95% CI 0.2–11.8).

Median follow-up time was 54.28 months (IQR 39.88, 59.17). The Low group had higher incidence of primary outcome, 16.1 events (95% CI 7.0–36.1) × 100 pts/year as compared with Medium (2.7 events 95% CI 1.3–6.0 × 100 pts/year) and High group (1.7 events 95% CI 0.2–11.8 × 100 pts/year) with a significant difference between rates (p = 0.003) (Fig. 2). There were no differences in the rate of immunosuppression regimens.

### Optimizing prognostic models for IgAN integrating serum C3 and C4

As reference models, we used Model-1 (M1), including the main clinical variables of CKD progression (age, gender, SBP, eGFR, uProt) and Model-3 (M3), including variables from the IntIgANpt (age, systolic and diastolic BP, eGFR, uProt, MEST-score). MEST-C parameter was not tested since not included in the tool. Model-2 (M2) and Model-4 (M4) were built by adding baseline C3 and C4 to M1 and M3, respectively. This enables us to explore the supplementary contribution of C3 and C4 to standardized models.

In M1 (Table 2), lower eGFR (OR 0.95, CI 0.92–0.99, p = 0.006) and higher uProt (OR = 1.39, CI 1.08–1.79, p = 0.008) were associated with higher risk of outcome, while there was not correlation with sex, age and SBP. In M2, higher uProt and lower eGFR still predicted a worse outcome, and both lower C3 (OR = 0.94, CI 0.89–0.94, p = 0.029) and higher C4 (OR 1.12, CI 1.01–1.25, p = 0.031) were associated to outcome. When the two models were compared, M2 showed lower values of both AIC (54.9 vs 64.1) and BIC (75.2 vs 81.3), higher discrimination (c-index 0.73 vs 0.63) and higher NR^2^ (67% vs 50%). LRT between M1 and M2 was significant (p-value = 0.003).

**Table 2:** Multivariate logistic regression models: correlation between basal characteristics and outcome with and without serum C3 and C4.

Characteristic	Model 1			Model 2		
	OR	95% CI	p-value	OR	95% CI	p-value
Age at biopsy (years)	0.99	0.94, 1.05	0.892	1.00	0.93, 1.06	0.957
Sex (M/F)	2.85	0.45, 17.7	0.261	2.11	0.19, 11.1	0.720
SBP (mmHg)	0.97	0.92, 1.01	0.394	0.98	0.92, 1.05	0.651
eGFR (ml/min/1.73m^2^)	0.95	0.92, 0.99	**0.006**	0.96	0.93, 0.99	**0.046**
uProt (g/24 h)	1.39	1.08, 1.79	**0.008**	1.51	1.10, 2.06	**0.009**
C3 (mg/dL)				0.94	0.89, 0.94	**0.029**
C4 (mg/dL)				1.12	1.01, 1.25	**0.031**
AIC	64.1			54.9		
BIC	81.3			75.2		
C-index	0.63			0.73		** < 0.001**
NR^2^	0.50			0.67		
LRT	**0.003**					

*SBP* systolic blood pressure, *eGFR* estimated glomerular filtration rate, *uProt* urinary protein, *OR* odds ratio, *CI* confidential interval, *AIC* Akaike information criterion, *BIC* Bayesian information criterion, *NR*^*2*^ Nagelkerke R^2^, *LRT* likelihood ratio test.

Significant values are given in bold.

In M3 (Table 3), variables from the IntIgANpt were used. uProt (OR 1.62, CI 1.12–5.21, p = 0.018) showed association with outcome. In M4, uProt (OR 2.32, CI 1.12–4.78, p = 0.022), age (OR 1.30, CI 1.01–1.56, p = 0.045) and C3 (OR 0.91, CI 0.83–0.99, p = 0.049) showed association with outcome. C4 had a positive relation with outcome though not statistically significant (OR 1.19, CI 0.99–1.45, p = 0.058). M4 had a lower AIC (39.9 vs 44.8) and BIC (66.0 vs 75.4) over M3, a higher c-index (0.77 vs 0.67) and NR^2^ (0.77 vs 0.68). LRT between M3 and M4 was significant (p = 0.009) testifying an improvement in prediction of M4.

**Table 3:** Multivariate logistic regression models: comparison of IntIgANpt variables with and without C3 and C4.

Characteristic	Model 3			Model 4		
	OR	95% CI	p-value	OR	95% CI	p-value
Age at biopsy (years)	1.62	0.92, 2.98	0.090	1.30	1.01, 1.56	**0.045**
SBP (mmHg)	0.78	0.59, 1.05	0.106	0.91	0.80, 1.05	0.231
DBP (mmHg)	0.93	0.66, 1.29	0.671	1.16	0.97, 1.50	0.225
eGFR (ml/min/1.73m^2^)	0.74	0.51, 0.98	**0.032**	0.96	0.91, 1.01	0.101
uProt (g/24 h)	1.62	1.12, 5.21	**0.018**	2.32	1.12, 4.78	**0.022**
MEST-M (0/1)	27.0	0.25, 282.78	**0.099**	52.0	0.85, 416.4	0.377
MEST-E (0/1)	0.60	0.08, 4.58	0.131	1.25	0.28, 25.3	0.926
MEST-S (0/1)	1.51	0.32, 71.5	0.089	2.92	0.14, 58.0	0.482
MEST-T						
1	4.87	0.13, 17.2	0.384	5.7	0.45, 10.8	0.710
2	3.10	0.34, 37.9	0.167	12.05	0.10, 34.4	0.613
C3 (mg/dL)				0.91	0.83, 0.99	**0.049**
C4 (mg/dL)				1.19	0.99, 1.45	0.058
AIC	44.8			39.9		
BIC	75.4			66.0		
C-index	0.67			0.77		** < 0.001**
NR^2^	0.68			0.77		
LRT	**0.009**					

*SBP* systolic blood pressure, *eGFR* estimated glomerular filtration rate, *uProt* urinary protein, *OR* odds ratio, *CI* confidential interval, *AIC* Akaike information criterion, *BIC* Bayesian information criterion, *NR*^*2*^ Nagelkerke R^2^, *LRT* likelihood ratio test.

Significant values are given in bold.

## Discussion

IgAN is a lifelong chronic affection which leads to a cumulative risk of poor renal outcome despite currently available treatments, with one-third of patients developing KF within 30 years after diagnosis^2^ and a 19% risk of recurrence after transplantation at 10 years^16^. This elicits the need for specific pathophysiology-oriented therapeutic strategies.

Despite being an autoimmune disease, the management of IgAN primarily relies on non-immunosuppressive treatment, such as lifestyle interventions, BP control, RAASi, SGLT2i and dual endothelin-angiotensin receptor antagonist. A course of steroid therapy is tipically reserved for individuals at high-risk of disease progression (i.e. uProt > 0.75 g/die) despite supportive treatment. Enrollment in clinical trials must be always considered in this patients^4,17–19^.

The CS is an integral component of the innate immune response, crucial for host defence against infections and tissue clearance from immunocomplexes or injured cells. Dysregulation of this system contributes to a wide spectrum of immune-mediated kidney diseases. Three primary activation pathways have been identified: classic pathway (CP), LP and AP^20^.

Complement-driven renal damage and its potential therapeutic targets is gaining increasing attention. Growing evidence indicates the pathogenetic role of complement activation, as evidenced by the presence of its components in mesangial immune complexes: more than 90% of kidney biopsies in IgAN show positive C3 staining in immunofluorescence, whereas C1q is rarely reported. This suggests potential activation of the AP and/or LP, rather than the CP. Deposition of complement regulatory proteins, such as properdin, factor-B and factor-H has also been described^21^.

Despite technical challenges and subjective measurement, the intensity of glomerular C3 staining might predict a higher risk of disease progression^22–24^. Moreover, a correlation between tissue deposits and disease activity was observed: the presence of factor-H related proteins such as CFHR5 -indicating AP activation- was associated with progressive disease, while C4d and mannose-binding lectin mesangial staining -indicating LP activation- was correlated with poorer outcomes^25–27^.

Further evidence for a complement-mediated pathogenesis was provided by genome-wide association studies, which highlighted the role of deletion in CFHR1-3 genes (positive regulator of AP) in IgAN susceptibility^28,29^. Endorsing this hypothesis, an overactivation of these factors, as indicated by elevated serum levels of CFHR1-5, has been associated to disease activity^30,31^.

Regardless of the site of production -systemically or locally- and the involved pathway -LP or AP-, aberrant complement activation by mesangial immunocomplexes precipitates glomerular injury and tissue inflammation. This occurs through production of anaphylatoxins (C3a, C5a) and cytolytic membrane attack complex (C5b9). Activation of coagulation cascade triggers further damage^20^.

Plasmatic C3 and C4 fractions are surrogate markers of complement activation and are widely measured in current nephrological practice. However, their role in IgAN prognosis is unclear and has yet to be elucidated. C3 values often fluctuate within the normal range, but lower levels have been correlated with poor kidney outcomes^32–35^. Some authors suggested that subjects with elevated serum IgA/C3 ratio are more likely to be diagnosed with IgAN and to exhibit a worst disease course^36–42^. Likewise, the galactose-deficient-IgA1/C3 ratio at the time of diagnosis has been suggested as independent predictor of CKD progression in a Chinese cohort^43^.

There are limited data regarding the role of serum C4 in IgAN pathogenesis and prognosis. In 2014, Zhu et al. reported that lower C4 levels at diagnosis correlated with worse outcome during follow-up, even though these individuals exhibited less severe lesions at the time of biopsy^44^. A more recent study conducted on 1356 Chinese IgAN patients indicated that serum C4 correlated positively with uProt and negatively with eGFR at baseline. Furthermore, survival analysis identified higher C4 levels as an independent risk-factor for disease progression. Noteworthy, C4 levels collected at the time of biopsy positively correlated with scoring of tubulointerstitial injury, glomerulosclerosis and crescents according to MEST-C score^13^. However, the aforementioned studies are limited because C4 was tested (or found to be statistically significant) individually, potentially biasing the contribution of the C3 component in determining renal prognosis.

Our data showed that the C3-hypocomplementemic subpopulation (< 90 mg/dl) had significant lower eGFR, lower C4 levels and higher proteinuria at baseline, suggesting a link between complement activation and more severe kidney injury at diagnosis. This was further confirmed by a significantly higher incidence rate of the primary composite kidney outcome in this group, regardless of immunosuppressant treatment.

The linear regression analyses highlighted a positive correlation between baseline C3 and C4 levels, as previously described by Bi et al., supporting the hypothesis of systemic complement consumption in complement-driven IgAN^13^. Furthermore, both C3 and C4 levels were found to be negatively correlated with serum IgM, pointing a role for IgM that deserves to be further investigation. This observation aligns with a recent study on 116 pediatric IgAN patients, which showed that mesangial IgM deposition at biopsy seems to be an independent risk factor for poor renal prognosis^45^.

Both C3 and C4 levels were significantly associated with baseline eGFR, confirming and expanding previous findings^13,14^.

Logistic regression models showed that the addition of C3 and C4 confers a better prediction accuracy as compared to reference models without these variables. Intriguingly, this was particularly true for goodness of fit analysis (LRT) and discrimination (c-index). These results suggest that incorporating complement levels into the baseline assessment of patients could help to discriminate those who are at higher risk of poor renal outcome. Additionally, the NR2 test indicates that serum complement enhances the percentage of events explained by the models, highlighting its inherent informativeness. Regardless of prognostic measures, another main finding of our study is that C3 and C4 levels were significantly associated with outcome despite the presence of robust variables in the models such as proteinuria and eGFR, that normally account for most of the prediction.

Our data validate previous observations by Pan et al. that decreased C3 and increased C4 levels are associated with poor renal prognosis in IgAN. These findings expand the evidence, for the first time to our knowledge, to a Caucasian-prevalent study population^14^. Additionally, our study has a longer follow-up period (54 vs 35 months) and a higher incidence of events (12.8% vs 9.8%) compared to the latter, despite smaller sample size (101 vs 403 patients) and harder endpoint (loss of 50% vs 30% of eGFR).

Although complement activation in IgAN is widely described in current literature, the relative contributions of AP and LP are neither clear nor easy to assess. Determining the dominant pathway driving IgAN progression may be useful to guide future tailored therapies.

In general, serum complement levels are influenced by the rate of their production and degradation. This balance varies depending on genetic factors (e.g., inherited C4 deficiency), systemic inflammation (i.e., acute phase reactants), the extent of complement activation, and the pathways through which this activation occurs. Despite the sources of variability mentioned above, it is reasonable to assume that serum levels of both C3 and C4 could provide an indication of complement activation status. Systemic hypocomplementemia suggests intra-renal inflammation, which correlates with poor kidney outcome.

As for the observed discordant correlation between lower C3 and higher C4 levels with an increased risk of outcome, we hypothesize that these results may partially reflect the dominant complement activation pathway. IgAN cases characterized by low-C3 and low-C4 might imply a prevalent activation of the LP (C4-mediated, *“lectin-pathic”*). Conversely, cases with low-C3 and high-C4 may indicate predominant AP activation (non-C4-mediated, *“altern-pathic”*), which seems to be associated with a poorer prognosis.

Similarly to what has been suggested in other complement-mediated kidney diseases, such as membranoproliferative glomerulonephritis^46^, adopting a “cluster-oriented” pathophysiological approach in IgAN could aid in addressing the current unmet medical need for biomarkers and risk stratification.

At present, serum complement C3 and C4 levels could be useful as markers to screen the more ‘*Complement-pathic*’ subset of patients in IgAN.

Integrating these markers into existing prognostic scores could enhance outcome prediction accuracy and optimize risk stratification in order to choose the best tailored therapies for our patients.

### Supplementary Information

Supplementary Information.

## Data Availability

The data that support the findings of this study are not openly available due to reasons of sensitivity and are available from the corresponding author upon reasonable request. Data are located in controlled access data storage at IRCCS Azienda Ospedaliero-Universitaria di Bologna, 40138 Bologna, Italy.

## Abbreviations

AIC: Akaike information criterion
AP: Alternative pathway
BIC: Bayesian information criterion
BP: Blood pressure
CKD: Chronic kidney disease
CS: Complement system
CP: Classical pathway
AP: Alternative pathway
LP: Lectin pathway
DBP: Diastolic blood pressure
eGFR: Estimated glomerular filtration rate
Ig: Immunoglobulin
IgAN: IgA nephropathy
IntIgANPT: International IgA nephropathy prediction tool
IQR: Interquartile range
KF: Kidney failure
LRT: Likelihood ratio test
NR^2^: Nagelkerke R square
RAASi: Renin–angiotensin–aldosterone system inhibitors
SBP: Systolic blood pressure
SD: Standard deviations
SGLT2i: Sodium/glucose cotransporter 2 inhibitors
uProt: 24H-hour urine protein

## References

[1] O'Shaughnessy MM, Hogan SL, Thompson BD, Coppo R, Fogo AB, Jennette JC (2018). Glomerular disease frequencies by race, sex and region: Results from the International Kidney Biopsy Survey. Nephrol. Dial Transplant..

[2] Lai KN, Tang SC, Schena FP, Novak J, Tomino Y, Fogo AB, Glassock RJ (2016). IgA nephropathy. Nat. Rev. Dis. Primers..

[3] Rodrigues JC, Haas M, Reich HN (2017). IgA nephropathy. Clin. J. Am. Soc. Nephrol..

[4] Rovin BH, Adler SG, Barratt J, Bridoux F, Burdge KA, Chan TM, Cook HT, Fervenza FC, Gibson KL, Glassock RJ, Jayne DRW, Jha V, Liew A, Liu ZH, Mejía-Vilet JM, Nester CM, Radhakrishnan J, Rave EM, Reich HN, Ronco P, Sanders JF, Sethi S, Suzuki Y, Tang SCW, Tesar V, Vivarelli M, Wetzels JFM, Lytvyn L, Craig JC, Tunnicliffe DJ, Howell M, Tonelli MA, Cheung M, Earley A, Floege J (2021). Executive summary of the KDIGO 2021 guideline for the management of glomerular diseases. Kidney Int..

[5] Bartosik LP, Lajoie G, Sugar L, Cattran DC (2001). Predicting progression in IgA nephropathy. Am. J. Kidney Dis..

[6] Tanaka S, Ninomiya T, Katafuchi R, Masutani K, Tsuchimoto A, Noguchi H, Hirakata H, Tsuruya K, Kitazono T (2013). Development and validation of a prediction rule using the Oxford classification in IgA nephropathy. Clin. J. Am. Soc. Nephrol..

[7] Chen T, Li X, Li Y, Xia E, Qin Y, Liang S, Xu F, Liang D, Zeng C, Liu Z (2019). Prediction and risk stratification of kidney outcomes in IgA nephropathy. Am. J. Kidney Dis..

[8] Barbour SJ, Coppo R, Zhang H, Liu ZH, Suzuki Y, Matsuzaki K, Katafuchi R, Er L, Espino-Hernandez G, Kim SJ, Reich HN, Feehally J, Cattran DC (2019). Evaluating a new international risk-prediction tool in IgA nephropathy. JAMA Intern Med..

[9] Provenzano M, Rotundo S, Chiodini P, Gagliardi I, Michael A, Angotti E, Borrelli S, Serra R, Foti D, De Sarro G, Andreucci M (2020). Contribution of predictive and prognostic biomarkers to clinical research on chronic kidney disease. Int. J. Mol. Sci..

[10] Maillard N, Wyatt RJ, Julian BA, Kiryluk K, Gharavi A, Fremeaux-Bacchi V, Novak J (2015). Current understanding of the role of complement in IgA nephropathy. J. Am. Soc. Nephrol..

[11] Roos A, Rastaldi MP, Calvaresi N, Oortwijn BD, Schlagwein N, van Gijlswijk-Janssen DJ, Stahl GL, Matsushita M, Fujita T, van Kooten C, Daha MR (2006). Glomerular activation of the lectin pathway of complement in IgA nephropathy is associated with more severe renal disease. J. Am. Soc. Nephrol..

[12] Seelen MA, Roos A, Daha MR (2005). Role of complement in innate and autoimmunity. J. Nephrol..

[13] Bi TD, Zheng JN, Zhang JX, Yang LS, Liu N, Yao L, Liu LL (2019). Serum complement C4 is an important prognostic factor for IgA nephropathy: A retrospective study. BMC Nephrol..

[14] Pan M, Zhang J, Li Z, Jin L, Zheng Y, Zhou Z, Zhen S, Lu G (2017). Increased C4 and decreased C3 levels are associated with a poor prognosis in patients with immunoglobulin A nephropathy: A retrospective study. BMC Nephrol..

[15] Tangri N, Grams ME, Levey AS (2016). Multinational assessment of accuracy of equations for predicting risk of kidney failure: A meta-analysis. JAMA.

[16] Uffing A, Pérez-Saéz MJ, Jouve T, Bugnazet M, Malvezzi P, Muhsin SA, Lafargue MC, Reindl-Schwaighofer R, Morlock A, Oberbauer R, Buxeda A, Burballa C, Pascual J, von Moos S, Seeger H, La Manna G, Comai G, Bini C, Russo LS, Farouk S, Nissaisorakarn P, Patel H, Agrawal N, Mastroianni-Kirsztajn G, Mansur J, Tedesco-Silva H, Ventura CG, Agena F, David-Neto E, Akalin E, Alani O, Mazzali M, Manfro RC, Bauer AC, Wang AX, Cheng XS, Schold JD, Berger SP, Cravedi P, Riella LV (2021). Recurrence of IgA nephropathy after kidney transplantation in adults. Clin. J. Am. Soc. Nephrol..

[17] Anders HJ, Peired AJ, Romagnani P (2022). SGLT2 inhibition requires reconsideration of fundamental paradigms in chronic kidney disease, 'diabetic nephropathy', IgA nephropathy and podocytopathies with FSGS lesions. Nephrol. Dial Transplant..

[18] Wheeler DC, Toto RD, Stefánsson BV, Jongs N, Chertow GM, Greene T, Hou FF, McMurray JJV, Pecoits-Filho R, Correa-Rotter R, Rossing P, Sjöström CD, Umanath K, Langkilde AM, Heerspink A (2021). A pre-specified analysis of the DAPA-CKD trial demonstrates the effects of dapagliflozin on major adverse kidney events in patients with IgA nephropathy. Kidney Int..

[19] Heerspink HJL, Radhakrishnan J, Alpers CE, Barratt J, Bieler S, Diva U, Inrig J, Komers R, Mercer A, Noronha IL, Rheault MN, Rote W, Rovin B, Trachtman H, Trimarchi H, Wong MG, Perkovic V (2023). Sparsentan in patients with IgA nephropathy: A prespecified interim analysis from a randomised, double-blind, active-controlled clinical trial. Lancet..

[20] Noris M, Remuzzi G (2013). Overview of complement activation and regulation. Semin. Nephrol..

[21] Roberts IS (2014). Pathology of IgA nephropathy. Nat. Rev. Nephrol..

[22] Wu D, Li X, Yao X, Zhang N, Lei L, Zhang H, Tang M, Ni J, Ling C, Chen Z, Chen X, Liu X (2021). Mesangial C3 deposition and serum C3 levels predict renal outcome in IgA nephropathy. Clin. Exp. Nephrol..

[23] Xie M, Zhu Y, Wang X, Ren J, Guo H, Huang B, Wang S, Wang P, Liu Y, Liu Y, Zhang J (2023). Predictive prognostic value of glomerular C3 deposition in IgA nephropathy. J. Nephrol..

[24] Wu J, Hu Z, Wang Y, Hu D, Yang Q, Li Y, Dai W, Zhu F, Yang J, Wang M, Zhu H, Liu L, He X, Han M, Yao Y, Pei G, Zeng R, Xu G (2021). Severe glomerular C3 deposition indicates severe renal lesions and a poor prognosis in patients with immunoglobulin A nephropathy. Histopathology..

[25] Medjeral-Thomas NR, Troldborg A, Constantinou N, Lomax-Browne HJ, Hansen AG, Willicombe M, Pusey CD, Cook HT, Thiel S, Pickering MC (2017). Progressive IgA nephropathy is associated with low circulating Mannan-binding lectin-associated serine protease-3 (MASP-3) and increased glomerular factor H-related protein-5 (FHR5) deposition. Kidney Int. Rep..

[26] Espinosa M, Ortega R, Sánchez M, Segarra A, Salcedo MT, González F, Camacho R, Valdivia MA, Cabrera R, López K, Pinedo F, Gutierrez E, Valera A, Leon M, Cobo MA, Rodriguez R, Ballarín J, Arce Y, García B, Muñoz MD, Praga M (2014). Association of C4d deposition with clinical outcomes in IgA nephropathy. Clin. J. Am. Soc. Nephrol..

[27] Segarra A, Romero K, Agraz I, Ramos N, Madrid A, Carnicer C, Jatem E, Vilalta R, Lara LE, Ostos E, Valtierra N, Jaramillo J, Arredondo KV, Ariceta G, Martinez C (2018). Mesangial C4d deposits in early IgA nephropathy. Clin. J. Am. Soc. Nephrol..

[28] Gharavi AG, Kiryluk K, Choi M, Li Y, Hou P, Xie J, Sanna-Cherchi S, Men CJ, Julian BA, Wyatt RJ, Novak J, He JC, Wang H, Lv J, Zhu L, Wang W, Wang Z, Yasuno K, Gunel M, Mane S, Umlauf S, Tikhonova I, Beerman I, Savoldi S, Magistroni R, Ghiggeri GM, Bodria M, Lugani F, Ravani P, Ponticelli C, Allegri L, Boscutti G, Frasca G, Amore A, Peruzzi L, Coppo R, Izzi C, Viola BF, Prati E, Salvadori M, Mignani R, Gesualdo L, Bertinetto F, Mesiano P, Amoroso A, Scolari F, Chen N, Zhang H, Lifton RP (2011). Genome-wide association study identifies susceptibility loci for IgA nephropathy. Nat. Genet..

[29] Kiryluk K, Li Y, Sanna-Cherchi S, Rohanizadegan M, Suzuki H, Eitner F, Snyder HJ, Choi M, Hou P, Scolari F, Izzi C, Gigante M, Gesualdo L, Savoldi S, Amoroso A, Cusi D, Zamboli P, Julian BA, Novak J, Wyatt RJ, Mucha K, Perola M, Kristiansson K, Viktorin A, Magnusson PK, Thorleifsson G, Thorsteinsdottir U, Stefansson K, Boland A, Metzger M, Thibaudin L, Wanner C, Jager KJ, Goto S, Maixnerova D, Karnib HH, Nagy J, Panzer U, Xie J, Chen N, Tesar V, Narita I, Berthoux F, Floege J, Stengel B, Zhang H, Lifton RP, Gharavi AG (2012). Geographic differences in genetic susceptibility to IgA nephropathy: GWAS replication study and geospatial risk analysis. PLoS Genet..

[30] Medjeral-Thomas NR, Lomax-Browne HJ, Beckwith H, Willicombe M, McLean AG, Brookes P, Pusey CD, Falchi M, Cook HT, Pickering MC (2017). Circulating complement factor H-related proteins 1 and 5 correlate with disease activity in IgA nephropathy. Kidney Int..

[31] Tortajada A, Gutiérrez E, Goicoechea de Jorge E, Anter J, Segarra A, Espinosa M, Blasco M, Roman E, Marco H, Quintana LF, Gutiérrez J, Pinto S, Lopez-Trascasa M, Praga M, Rodriguez de Córdoba S (2017). Elevated factor H-related protein 1 and factor H pathogenic variants decrease complement regulation in IgA nephropathy. Kidney Int..

[32] Le Stang MB, Gleeson PJ, Daha MR, Monteiro RC, van Kooten C (2021). Is complement the main accomplice in IgA nephropathy? From initial observations to potential complement-targeted therapies. Mol. Immunol..

[33] Kim SJ, Koo HM, Lim BJ, Oh HJ, Yoo DE, Shin DH, Lee MJ, Doh FM, Park JT, Yoo TH, Kang SW, Choi KH, Jeong HJ, Han SH (2012). Decreased circulating C3 levels and mesangial C3 deposition predict renal outcome in patients with IgA nephropathy. PLoS One..

[34] Wyatt RJ, Kanayama Y, Julian BA, Negoro N, Sugimoto S, Hudson EC, Curd JG (1987). Complement activation in IgA nephropathy. Kidney Int..

[35] Zwirner J, Burg M, Schulze M, Brunkhorst R, Götze O, Koch KM, Floege J (1997). Activated complement C3: A potentially novel predictor of progressive IgA nephropathy. Kidney Int..

[36] Tomino Y, Suzuki S, Imai H, Saito T, Kawamura T, Yorioka N, Harada T, Yasumoto Y, Kida H, Kobayashi Y, Endoh M, Sato H, Saito K (2000). Measurement of serum IgA and C3 may predict the diagnosis of patients with IgA nephropathy prior to renal biopsy. J. Clin. Lab. Anal..

[37] Maeda A, Gohda T, Funabiki K, Horikoshi S, Shirato I, Tomino Y (2003). Significance of serum IgA levels and serum IgA/C3 ratio in diagnostic analysis of patients with IgA nephropathy. J. Clin. Lab. Anal..

[38] Yanagawa H, Suzuki H, Suzuki Y, Kiryluk K, Gharavi AG, Matsuoka K, Makita Y, Julian BA, Novak J, Tomino Y (2014). A panel of serum biomarkers differentiates IgA nephropathy from other renal diseases. PLoS One..

[39] Gong WY, Liu M, Luo D, Liu FN, Yin LH, Li YQ, Zhang J, Peng H (2019). High serum IgA/C3 ratio better predicts a diagnosis of IgA nephropathy among primary glomerular nephropathy patients with proteinuria ≤ 1 g/d: An observational cross-sectional study. BMC Nephrol..

[40] Zhang J, Wang C, Tang Y, Peng H, Ye ZC, Li CC, Lou TQ (2013). Serum immunoglobulin A/C3 ratio predicts progression of immunoglobulin A nephropathy. Nephrology (Carlton)..

[41] Komatsu H, Fujimoto S, Hara S, Sato Y, Yamada K, Eto T (2004). Relationship between serum IgA/C3 ratio and progression of IgA nephropathy. Intern Med..

[42] Stefan G, Stancu S, Boitan B, Zugravu A, Petre N, Mircescu G (2020). Is there a role for IgA/C3 ratio in IgA nephropathy prognosis? An outcome analysis on an European population. Iran J. Kidney Dis..

[43] Chen P, Yu G, Zhang X, Xie X, Wang J, Shi S, Liu L, Lv J, Zhang H (2019). Plasma galactose-deficient IgA1 and C3 and CKD progression in IgA nephropathy. Clin. J. Am. Soc. Nephrol..

[44] Zhu B, Zhu CF, Lin Y, Perkovic V, Li XF, Yang R, Tang XL, Zhu XL, Cheng XX, Li Q, Chen HY, Sun Y, Chen QW, Wang YJ (2015). Clinical characteristics of IgA nephropathy associated with low complement 4 levels. Ren Fail..

[45] Xiong L, Liu L, Tao Y, Guo H (2023). Clinical significance of IgM and C3 deposition in children with primary immunoglobulin A nephropathy. J. Nephrol..

[46] Iatropoulos P, Daina E, Curreri M, Piras R, Valoti E, Mele C, Bresin E, Gamba S, Alberti M, Breno M, Perna A, Bettoni S, Sabadini E, Murer L, Vivarelli M, Noris M, Remuzzi G (2018). Cluster analysis identifies distinct pathogenetic patterns in C3 glomerulopathies/immune complex-mediated membranoproliferative GN. J. Am. Soc. Nephrol..

