# Supplementary material for: Role of serum complement C3 and C4 on kidney outcomes in IgA nephropathy
Source: Sci Rep. 2024 Jul 13;14:16224. doi: 10.1038/s41598-024-65857-w (PMC11246413; doi:10.1038/s41598-024-65857-w)

Table 1 - Supplementary. Multivariate linear regression between serum C3 and C4 with eGFR at baseline.

Characteristic	Beta	p-value
C3 (mg/dL)	0.30	0.05
C4 (mg/dL)	-0.76	0.04

Figure 1 – Supplementary. Correlations between C3 and C4 with IgA, IgG.

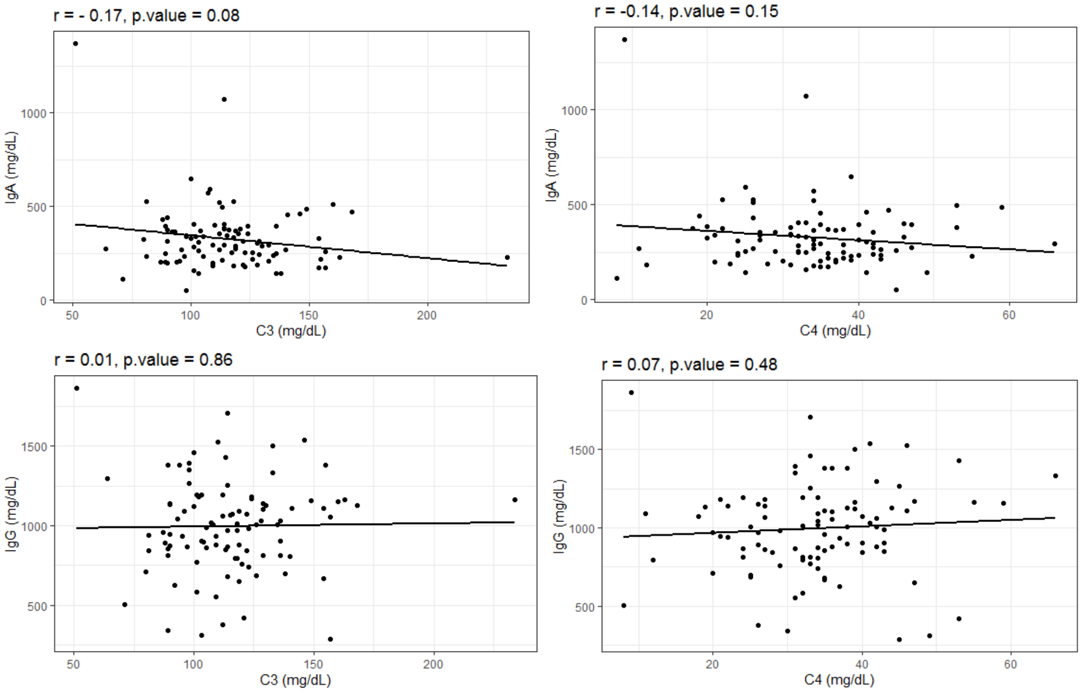

Supplement: Supplementary file 1 — Supplementary Information. [file 41598_2024_65857_MOESM1_ESM.pdf]
